# Supplementary material for: Quality Evaluation of Atractylodis Macrocephalae Rhizoma Based on Combinative Method of HPLC Fingerprint, Quantitative Analysis of Multi-Components and Chemical Pattern Recognition Analysis
Source: Molecules. 2021 Nov 25;26(23):7124. doi: 10.3390/molecules26237124 (PMC8658834; doi:10.3390/molecules26237124)
Supplement: Supplementary file 1 [file molecules-26-07124-s001.zip › molecules-1432204-supplementary.pdf]

Supplementary Material

Quality Evaluation of Atractylodis Macrocephalae Rhizoma Based on Combinative Method of HPLC Fingerprint, Quantitative Analysis of Multi-Components and Chemical Pattern Recognition Analysis

Cheng Zheng, Wenting Li, Yao Yao and Ying Zhou *

NMPA Key Laboratory of Quality Evaluation of Traditional Chinese Medicine (Traditional Chinese Patent Medicine), Zhejiang Institute for Food and Drug Control, Hangzhou 310052, China;
zhengcheng@zjyj.org.cn (C.Z.); liwenting@zjyj.org.cn (W.L.); yaoyao@zjyj.org.cn (Y.Y.)
* Correspondence: zhouying@zjyj.org.cn; Tel.: +86-(0571)-86459414

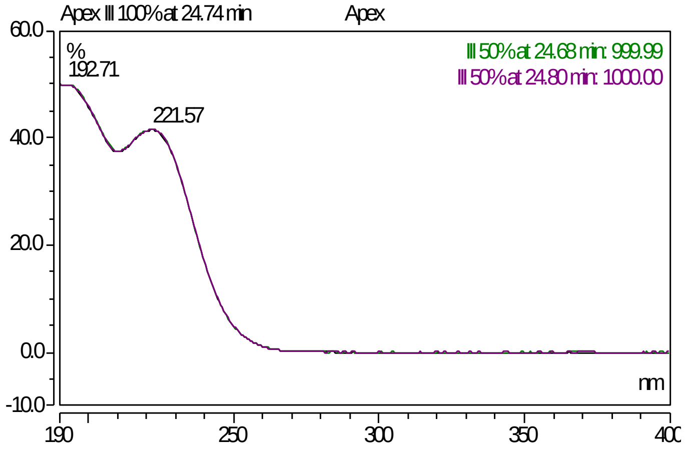

Figure S1.the spectra of the standard of atractylenolide III

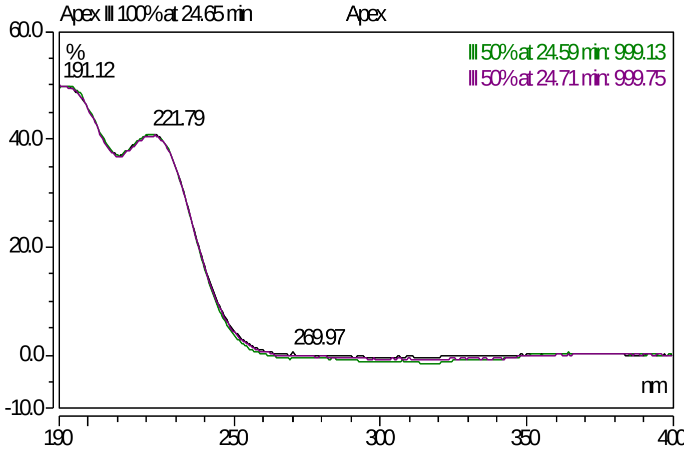

Figure S2. the spectra of the component of atractylenolide III

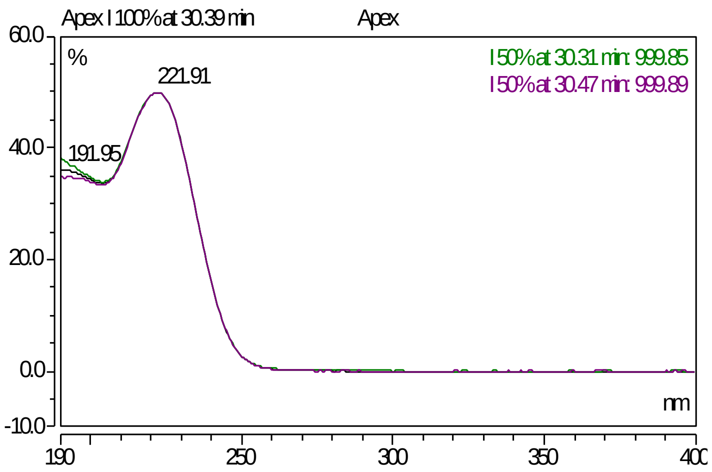

Figure S3.the spectra of the standard of atractylenolide II

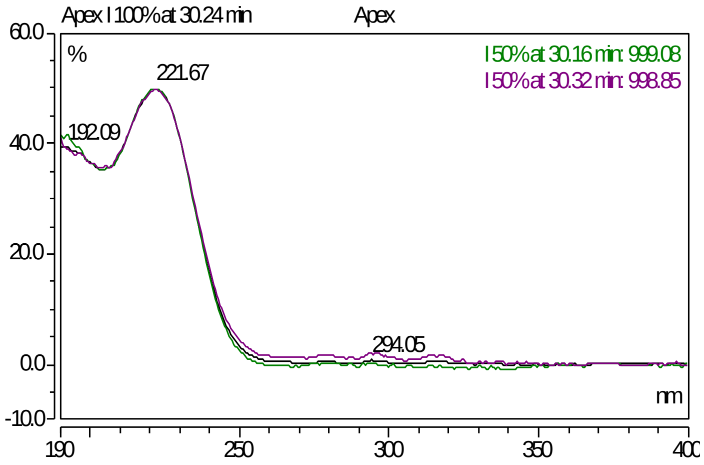

Figure S4. the spectra of the component of atractylenolide II

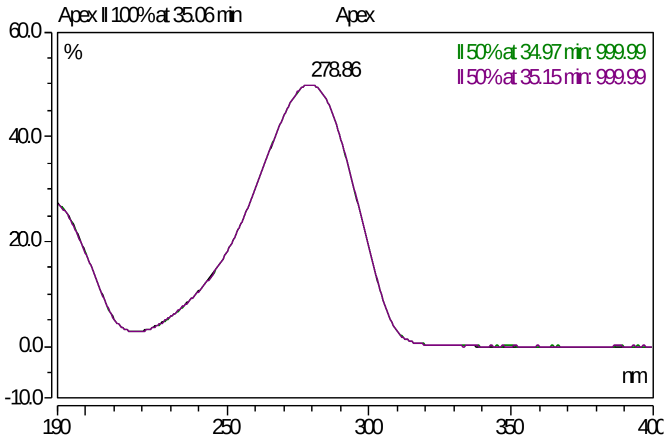

Figure S5.the spectra of the standard of atractylenolide I

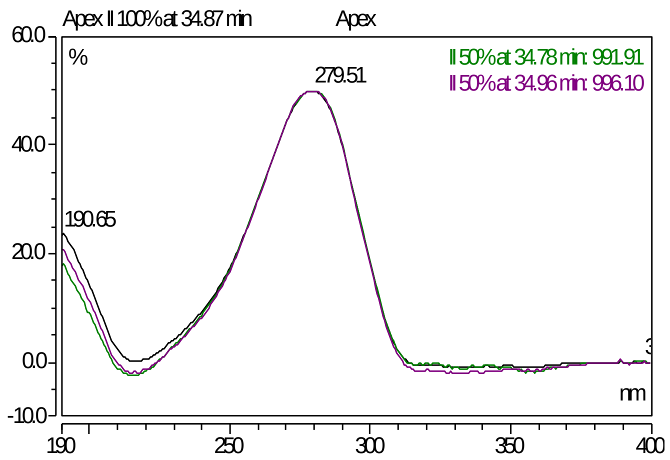

Figure S6. the spectra of the component of atractylenolide I

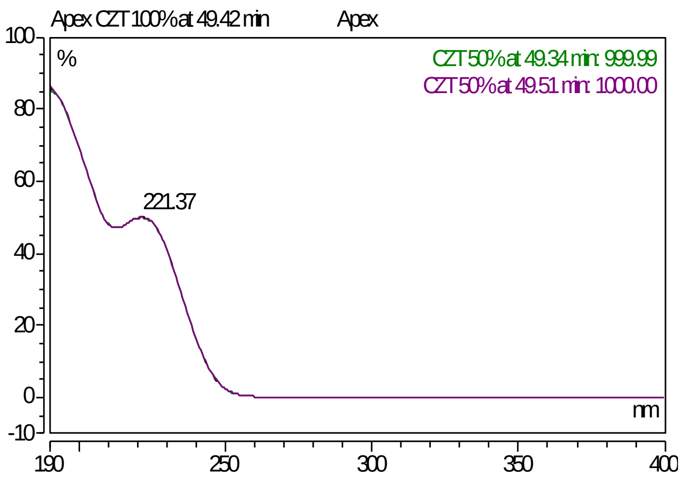

Figure S7.the spectra of the standard of atractylone

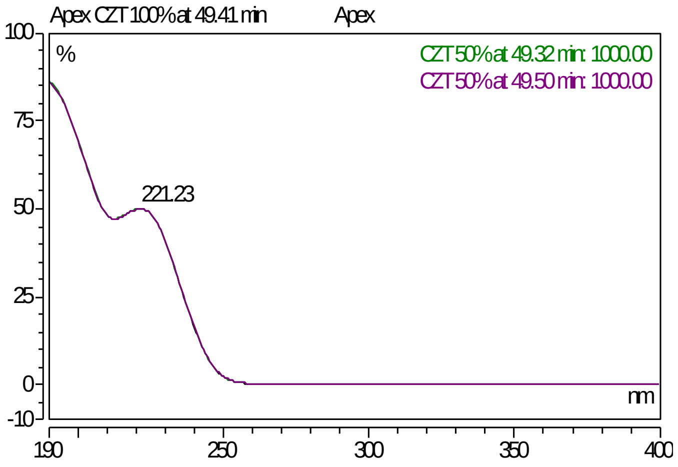

Figure S8. the spectra of the component of atractylone
